# What's new in Emergencies, Trauma and Shock? Coagulation is in the focus!

**DOI:** 10.4103/0974-2700.58649

**Published:** 2010

**Authors:** Max Ragaller

**Affiliations:** Department of Anesthesiology and Intensive Care Medicine, University Clinic Carl Gustav Carus, Dresden, Germany

Uncontrolled bleeding and life-threatening coagulopathy are common clinical but unresolved problems in trauma patients as well as in major orthopedic surgery.

As a consequence of major blood loss, a fundamental impairment of hemostasis due to plasmatic coagulation disturbance as well as platelet dysfunction will appear. Development of a substantial coagulopathy after major bleeding is associated with an increase in morbidity and mortality. Beside severe brain injury, uncontrolled bleeding with coagulopathy is the second most common cause of death in trauma victims.[1–4] Furthermore, at least in industrialized countries, a lot of patients were prophylactic or therapeutically on anticoagulants due to cardiovascular diseases, which may influence blood loss as well as the coagulation system in general.

Coagulopathy associated with traumatic injury is the result of multiple independent but interacting mechanisms and involves all components of the hemostatic system. In brief, tissue injury initiates coagulation as endothelial damage leads to exposure of tissue factor (TF) and subendothelial type III collagen, which bind von Willebrand factor, platelets, and activated factor VII. This complex activates several plasma coagulation proteases, which generate thrombin and ending in fibrin clot formation. Starting with a small amount of TF, this amplification process leads to a large fibrin production, which is in combination with platelets usually adequate for cessation of bleeding.[4–6]

In trauma and major surgical patients, *dilution coagulopathy* with a loss of coagulation factors due to major bleeding, large wounds, and tissue damage in combination with the rapid infusion of large amounts of colloids and crystalloids might be the most frequent coagulopathy. In this context, fibrinogen consumption with an incremental lack of fibrinogen is the first measurable hit to the coagulation system.[6–8] Additionally the use of colloids like HES, gelatins, or dextrans will dilute the plasmatic as well as corpuscular components of the coagulation system. Furthermore all colloids will dose dependently interfere with fibrin polymerization, which results in a reduction of clot stability.[9,10]

Secondly, there is evidence that a *systemic anticoagulation via the protein-C pathway* is activated and contributes substantially to the early coagulopathic state in trauma victims. Impaired tissue perfusion due to traumatic shock triggers increased expression of thrombmodulin and protein C resulting in a general anticoagulation effect.[4,11,12]

Thirdly, *hyperfibrinolysis* might also be an important issue in trauma patients due to the endothelial injury because of a substantial release of tissue plasminogen activator (tPA). Fibrinolysis furthermore exacerbates by the combined effect of endothelial tPA release following ischemia and the inhibition of plasminogen activator inhibitor-1 (PAI-1) in shock. Recent findings suggest that hyperfibrinolysis may be the dominant reason for the increased bleeding in brain-injured patients.[4,12] Due to diagnostic difficulties of hyperfibrinolysis, this pathology will probably appear more often than diagnosed, especially in patients with ongoing bleeding despite a massive transfusion of blood products. At the moment, thromboelastography is the only reliable method for diagnosing hyperfibrinolysis.[6,11,13]

Fourth, *disseminated intravascular coagulation (* DIC) as a very complex impairment of the coagulation system will contribute to the microcirculatory dysfunction and multiple organ failure. Sepsis, severe trauma, or massive transfusion especially in the later phase during ICU-stay induce a general and diffuse clot formation in the microcirculation, which results in a decrease in the functional microvascular density of more than 50%. This contributes to a fundamental tissue hypoxia and consecutively to multiple organ failure and death.[14]

Fifth, some predisposing factors or conditions like hypothermia, acidosis, and anemia will substantially embarrass the coagulation potential.[4,9] For example, only a slight hypothermia of 35°C leads to a significant increase in the intraoperative blood loss due to impaired plasmatic coagulation as well as platelet adhesion and aggregation. Platelets are probably more sensitive to hypothermia by a substantial reduction in von Willebrand factor traction on glycoprotein Ib/IX, which is completely absent below 30°C. Acidosis below 7.1 pH induces a nearly complete loss of factor VIIa activity.[15] Furthermore, the impact of red blood cells for a successful coagulation has been underscored in clinical praxis since it became obvious that a substantial number of red blood cells are necessary for ADP release and to push the platelets near the vessel wall to induce clot formation.[16]

Keeping all this issues in mind, rapid diagnostic tools are necessary to provide a goal-oriented and effective therapeutic strategy to improve the prognosis of major hemorrhage.

The correct analysis of the coagulation system in the situation of major bleeding is still a matter of debate, and the optimal diagnostic has not been found up to now.[17] There are at first the classical screening coagulation tests like PT or INR, aPTT, TT, fibrinogen, and the number of platelets. These tests usually performed at 37°C are time-consuming (at least 30 min) and usually performed in the hemostasis laboratory. These parameters may have their eligibility in surgical patients, but due to the unpredictable dynamics of a major hemorrhage, diagnostic tools at the beside (point-of-care diagnostics) became more important. Techniques like thromboelastography or rotation thromboelastography (ROTEM™) are now available at the bedside (point of care devices). Parameters like coagulation time (CT), clot formation time (CFT), maximum clot firmness (MCF), and lysis index (LI) will give valuable information about clot formation and hyperfibrinolysis in a few minutes (10–15 min).[6,13] Due to these fast and valuable results of the viscoelastic measurements at the bedside, aggressive treatment of hyperfibrinolysis and factor deficiency should enable a more effective correction of coagulopathy.

Relating to this broad complex of impaired coagulation in trauma patients, Kanchana and co-workers present a clinical study named “Coagulation Studies in Patients with Orthopaedic Injuries” in this issue of JETS.

The prospective study enrolled 48 orthopedic trauma patients without brain injury (GCS>13) over a period of 6 months. The authors correlated the DIC-score as described by Taylor with the well-known injury severity score (ISS). Interestingly, the DIC score did not correlate with the ISS positively and only a group of 10 patients (20%) did have a mild increased DIC-score on admission despite an ISS of 34 in median. This is in contrast to several other earlier trials. However, this might be a consequence of the exclusion of brain trauma and to the fact that none of the enrolled patients had requirement of massive blood transfusion. Unfortunately the amounts of transfused units of red blood cells or fresh frozen plasma as well as fibrinogen are not given in detail. Furthermore, as pointed out by Fries *et al.* recently, coagulopathy in trauma patients is different to DIC at least in the early phase.[6] Another surprising result of the study is the increase in fibrinogen levels over time beginning from the admission until postoperatively. This is again discordant to previous trails in severe multiple trauma patients, in which an early and profound reduction in fibrinogen levels is usually found.[6–8] The absence of information relating the amount of transfused blood products during surgery makes these data difficult to interpret. One might speculate that the injuries as well as the blood loss were not that severe in these patients and, therefore, fibrinogen increases as acute phase protein over time.[18] Another explanation of this intriguing finding might be a sufficient transfusion therapy. However, as pointed out above detailed information regarding the blood and plasma replacement therapy is not given in this paper.

In conclusion, the current paper adds new but pretty surprising facts to the topic of coagulation management after severe trauma. As pointed out by the authors, more and larger clinical trials with a careful monitoring of the coagulation system are necessary to evaluate the impact of their results on the management of severely injured patients.

Putting all the evidence relating coagulopathy following severe hemorrhage together, a simplified therapeutic approach could be given as pointed out in Table 1.

**Table 1:** Diagnostic and therapeutic algorithm of coagulopathy in severe hemorrhage (emergency department, operation theatre or ICU)

Diagnosis	Therapy	Co-factors
Goals of coagulation management	1. Optimize clot formation (Fibrinogen 4-6g or FFP 20-40 ml/kg bw.)	1. Keep Hb about 7-10 mg/dl or Ht. 30±3 %
Clinical stop of bleeding in general!	Platelets if < 50,000 G/l	2. Achieve normothermia 36–37.5°C
	2 Optimize plasmatic Coagulation	
Parameters to control repeatedly	(FFP 20-30ml/kg bw. or PPSB 20-40IU/kg bw.)	3. Resolve acidosis pH> 7.20
Fibrinogen > 200 mg/dl or 2.0 g/l	3. Hyperfibrinolysis?	
Platelets: 50-80.000G/l aPTT < 50 sec	(Tranexamic acid 10-20mg/kg bw.)	4. Restrict colloids if possible
PT > 50% (INR < 1,5) if available thrombelastography	4. Think of single Factors Factor VIIa (90μg/kg bw.)	
	Factor XIII (20 IU/kg bw.)	
